# Coating Medpor^®^ Implant with Tissue-Engineered Elastic Cartilage

**DOI:** 10.3390/jfb11020034

**Published:** 2020-05-22

**Authors:** Dong Joon Lee, Jane Kwon, Yong-Il Kim, Yong Hoon Kwon, Samuel Min, Hae Won Shin

**Affiliations:** 1 Oral and Craniofacial Health Science Institute, School of Dentistry, University of North Carolina, CB #7454, Chapel Hill, NC 27599, USA; jane_kwon@med.unc.edu (J.K.); kimyongil@pusan.ac.kr (Y.-I.K.); y0k0916@pusan.ac.kr (Y.H.K.); sammin35@gmail.com (S.M.); 2 Department of Orthodontics, Dental Research Institute, Pusan National University Dental Hospital, Geumoro 20, Mulgeum, Yangsan 50612, Korea; 3 Department of Dental Materials, Pusan National University, Busandaehak-ro 49, Mulgeum, Yangsan 50612, Korea; 4 Department of Neurology, School of Medicine, University of North Carolina, CB #7025, Chapel Hill, NC 27599, USA

**Keywords:** poly(DL-lactic-co-glycolic acid), high-density porous polyethylene (Medpor^®^), chondrocytes, scaffolds, neocartilage

## Abstract

Inert biomaterials used for auricular reconstruction, which is one of the most challenging and diverse tasks in craniofacial or head and neck surgery, often cause problems such as capsule formation, infection, and skin extrusion. To solve these problems, scaffold consisting of inert biomaterial, high-density polyethylene (Medpor^®^) encapsulated with neocartilage, biodegradable poly(DL-lactic-co-glycolic acid) (PLGA) was created using a tissue engineering strategy. PLGA scaffold without Medpor^®^ was created to serve as the control. Scaffolds were vacuum-seeded with rabbit chondrocytes, freshly isolated from the ear by enzymatic digestion. Then, cell-seeded scaffolds were implanted subcutaneously in the dorsal pockets of nude mice. After 12 weeks, explants were analyzed by histological, biochemical, and mechanical evaluations. Although the PLGA group resulted in neocartilage formation, the PLGA–Medpor^®^ group demonstrated improved outcome with the formation of well-surrounded cartilage around the implants with higher mechanical strength than the PLGA group, indicating that Medpor^®^ has an influence on the structural strength of engineered cartilage. The presence of collagen and elastin fibers was evident in the histological section in both groups. These results demonstrated a novel method of coating implant material with engineered cartilage to overcome the limitations of using biodegradable scaffold in cartilage tissue regeneration. By utilizing the patient’s own chondrocytes, our proposed method may broaden the choice of implant materials while minimizing side effects and immune reaction for the future medical application.

## 1. Introduction

The loss of normal ear morphology due to congenital defect (microtia), trauma, or cancer requires a reconstructive surgery. Specifically, auricle is an outer most part of auditory system that can be easily damaged by external force because of its thin structure. To repair damaged ear, the framework for architectural support has to precede [1]. The ear framework is mainly composed of elastic cartilage, which is histologically similar to hyaline cartilage. Ear cartilage is surrounded by a connective tissue, perichondrium, which contains capillaries that supply oxygen and nutrients by diffusion into the matrix [2]. The ear cartilage consists of cells and extracellular matrices (ECMs). The major cell type is chondrocyte, which fill small lacunar spaces. The ECMs are mainly made of glycosaminoglycan (GAG), collagen (mostly type II), and elastin. The contents of GAG and the resistance to tension of the collagen and elastic fibers content have an effect on the resistance to compression and the viscoelasticity. These components in the matrix play a key role in providing flexibility to sustain against repeated bending [3].

Several materials, such as cartilaginous, osseous, and alloplastic have been reported as potential alternatives for the auricle reconstruction since 1891 [4,5]. Among those materials, autogenous costal cartilage and silicone have been the popular choices for ear reconstruction [6,7,8,9]. Although the autogenous rib cartilage grafts still remain as the gold standard for auricle reconstruction [10], their can cause major complications including donor site morbidity and creation of a second operation site in addition to their limited availability and the increased cost of harvest. Also, as such grafts rely heavily on the surgeon’s hand skill, their shapes can lack in precision. Most importantly, the rib cartilage grafts lead to gradual and progressive absorption [11,12].

Advancements of materials in medical application enabled variety of implantable substitutes to replace damaged human tissues. However, many artificially manufactured biomaterials cause inflammatory reaction, fibrosis by fibroblasts, infection, skin necrosis, extrusion, and more after implantation. To date, silicone and polyethylene have been most widely used as permanent tissue substitutes because of their reasonable biocompatibilities. Silicone and polyethylene are also the most popular implants for cartilage reconstruction. However, silicones do not become integrated into the surrounding tissues and cause a high rate of extrusion through the skin and infection [13]. Previous study showed that silicone implants were freely mobile within soft tissue and failed to interact with the host tissue [14]. The efficacy of high-density porous polyethylene was tested as a framework for ear reconstruction, and implants of different thickness placed in primate ear was tested as substitutes for the ear cartilage [13]. Its result was progressive compared to cases using silicone, but still limited in effectiveness. 

Recently, the technique to cover polyethylene implant with cartilage tissue using gel type of polymer such as Pluronic and fibrin was proposed [15,16]. However, the initial weakness of those hydrogels made the structure vulnerable to interruptions and damages by external forces before tissue formation. In addition, failure to control the thickness of cartilage formation and the efficiency of coverage presented major obstacles in proceeding to further applications.

**Figure 1:**
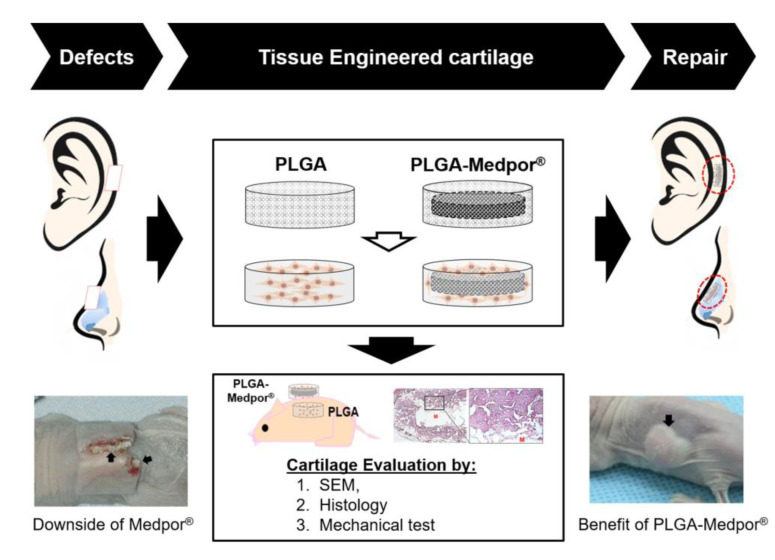
Scheme of the study. PLGA: poly(DL-lactic-co-glycolic acid) and Scanning electron microscopy (SEM).

To overcome these limitations, we have developed a porous poly(DL-lactic-co-glycolic acid) (PLGA)-coated Medpor^®^ scaffold seeded with isolated chondrocytes for auricular cartilage tissue regeneration (Figure 1). Medpor^®^ is a highly effective auricle implant allograft that has been used as surgical implant because of its similar properties to cartilage. By simply immersing Medpor^®^ in hot saline, its auricle contour can be reformed to the desired shape for ear reconstruction [17]. By manipulating two clinically popular biomaterials, PLGA and Medpor^®^, we have investigated the feasibility of those materials as substitutable implant in vivo. A complete coverage with cartilage tissue has the potential to improve Medpor^®^’s performance as an implant by preventing extreme inflammatory response that expels Medpor^®^ through skin.

## 2. Results

### 2.1. Chondrocyte Isolation and Collagen Type II Expression

**Figure 2:**
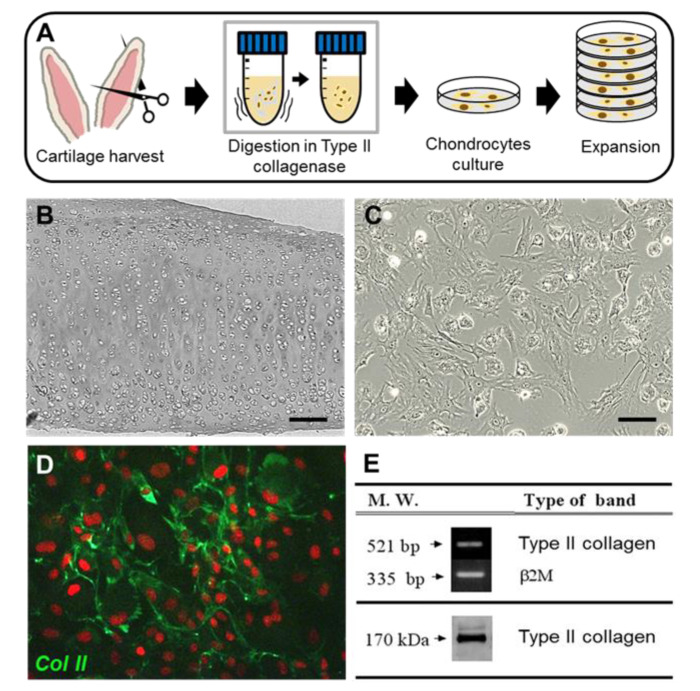
Cartilage tissues were isolated from rabbit ear, digested of matrices in collagenase type II solution, and expanded in chondrocytes monolayer culture (**A**). Rabbit ear cartilage, scale bar: 200 μm (**B**); chondrocytes, scale bar: 20 μm (**C**); immunostained chondrocytes at P4 with anti-collagen type II antibody conjugated with FITC (**D**); and band expression of type II collagen Ge4ne and protein by PCR and Western blot, respectively (**E**).

Chondrocytes were successfully isolated from rabbit ear cartilage for monolayer culture. Cells were expanded by subculture (Figure 2A) and tested for their specific marker, type II collagen, before the scaffold seeding. Chondrocytes at passages 4 were used for all experiments. The type II collagen gene was highly expressed in the isolated chondrocytes, and a high protein level of collagen type II secretion was also detected by Western blot analysis in the culture media (Figure 2E). Immunostaining of the chondrocyte culture showed positive expression against anti-collagen type II polyclonal antibody (ThermoFisher Scientific, Waltham, MA, USA), as clearly indicated by green FITC expression. The nuclei was stained with propedium idodie (PI) as red (Figure 2D).

### 2.2. PLGA-Medpor^®^ Scaffold

**Figure 3:**
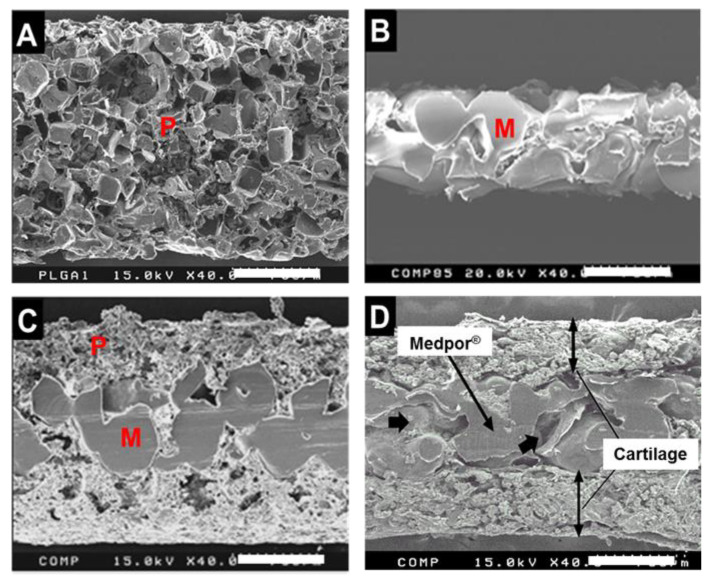
Scanning electron microscopy of the cross section of PLGA, shown as P (**A**); Medpor^®^, shown as M (**B**); PLGA–Medpor^®^ (**C**); and neocartilage regenerated with rabbit chondrocytes and Medpor^®^ (**D**). Scale bar: 750 µm.

Scanning electron microscopy was used to observe the cross section of the porous PLGA. The pores were somewhat irregular in shape, but were evenly distributed with highly interconnective morphology (Figure 3). In Figure 3, the Medpor also presented spaces among particles that adhered to each other, and PLGA was well integrated through the pores and surface on the top and bottom of Medpor in the PLGA–Medpor scaffold. Later, the interconnected porous structure of both PLGA and Medpor promoted efficient cell seeding and cartilage tissue ingrowth through pores and creation of a solid, well-combined cartilage tissue and Medpor^®^ structure (Figure 3).

### 2.3. In Vivo Cartilage Formation

**Figure 4:**
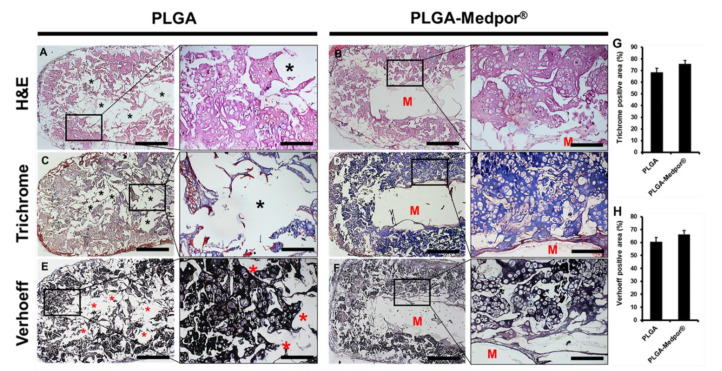
Histological analysis of cross-sectioned neocartilage with PLGA and PLGA–Medpor^®^. Collagen was stained blue by trichrome staining (**C**,**D**), prominent elastin expression was detected by Verhoeff staining in both neocartilages (**E**,**F**), and overall distribution of neocartilage was shown by H&E staining (**A**,**B**). Scale bars: 500 µm (left images, 4×) and 2.5 mm (right images, 20×). Quantification of collagen and elastin by ImageJ analysis (**G**,**H**); *n* = 3, * *p* > 0.05.

After 12 weeks of implantation in nude mice, neither sign of extrusion through the skin nor infection around the implanted area was observed. Gross examination on explants did not show much difference between the cartilage tissues made with either PLGA or PLGA–Medpor^®^. Both cartilage tissues demonstrated a solid morphology with milky color, which is similar to the morphology of natural rabbit ear cartilage. The H&E staining of neocartilages in both groups demonstrated that the chondrocytes were similar in morphology to those in native cartilage, with the cells located within lacunae, surrounded by cartilaginous matrix (Figure 4A,B). The neocartilage made of PLGA resulted in disintegrated tissue formation with the presence of many empty spaces (marked as *) in the center part of the neocartialge, whereas neocartilage with PLGA–Medpor^®^ showed formation of dense cartilage tissue around the Medpor^®^ and tight interface between the neocartilage and Medpor^®^ with ingrowth through the pores in the Medpor^®^. The cartilaginous matrices of neocartilage were intensively stained by Trichrome (Figure 4C,D) and Verhoeff staining (Figure 4E,F) for the secreted collagen (blue) and elastin fibers (black), respectively. PLGA–Medpor^®^ resulted in more prominent collagen and elastin expressions compared to the PLGA group.

### 2.4. Mechanical Analysis

**Figure 5:**
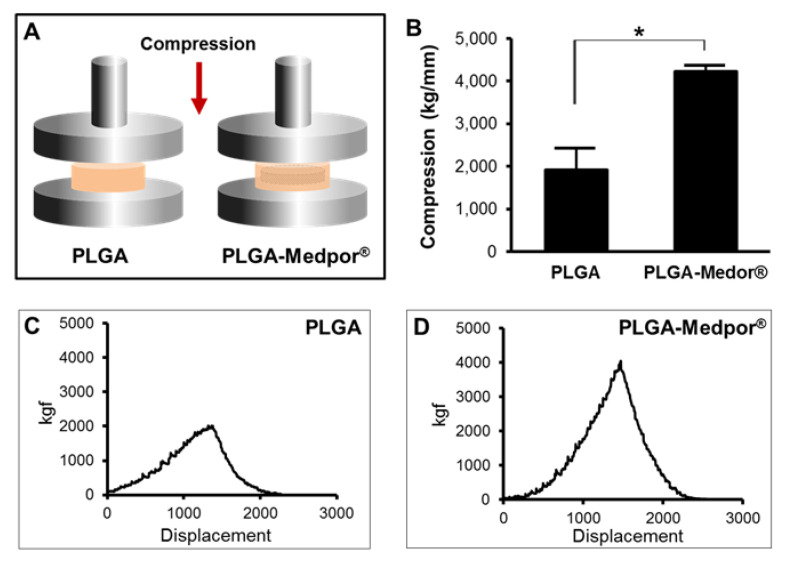
Compressive strengths of neocartilage with PLGA and PLGA–Medpor^®^ after 12 weeks of implantation (**A**,**B**); *n* = 5, * *p* < 0.05. Representative stress and strain curve patterns of neocartilages with PLGA (**C**) and PLGA–Medpor^®^ (**D**).

After 12 weeks of implantation, explants were measured of the pattern of load and displacement (Figure 5C,D). The mean values of the compressive strength were 1915.25 ± 506.34 and 4230.8 ± 139.24 kgf for neocartilage with PLGA and PLGA–Medpor^®^, respectively. The peak compressive strength of the PLGA–Medpor^®^ group was 2.21 times higher than that of the PLGA group (Figure 5B), indicating that Medpor^®^ has an influence on the structural strength of neocartilage.

## 3. Discussion and Conclusions

Medpor^®^ implant is manufactured from linear high-density polyethylene with interconnecting open pores, which enable rapid host tissue ingrowth. Because polyethylene is rigid in structure, Medpor^®^ has been used to mainly substitute hard and bony tissues. However, when Medpor^®^ is placed under thin skin, there is a high risk of exposure or extrusion due to complications including infection, inflammation, or foreign body reaction [18,19]. Applying any repetitive physical force on the site of implantation is detrimental as well. To solve the limit, we investigated the feasibility of using PLGA scaffold to coat Medpor^®^ implants using a tissue engineering strategy.

Cartilage tissue engineering is a rapidly developing area in research that aims to develop biological implants to repair or replace deformed or defective cartilage tissue in the body. However, the major disadvantage of the conventional approach to engineer cartilage using biodegradable polymers is the weaker mechanical strength. The absence of a control mechanism between degradation and matrix formation yields an incomplete or disintegrated cartilage tissue formation, which causes a deficiency of appropriate mechanical properties as natural cartilage [20]. Recently, the paradigm of scaffold materials for cartilage tissue engineering has shifted from single biodegradable polymer to multilayer structure, mixed with biodegradable and bioinert polymers. This approach allows the engineered cartilage tissue to overcome the limitation of implant materials in terms of physical property, foreign body reaction, skin protrusion, irritation, and more.

Although regenerating cartilage tissue from its specific chondrocyte without foreign material would be ideal as a non-immunogenic implant, this approach may lack the mechanical strength. Thus, multilayer approach may be considered as a practical solution due to certain unique advantages: (1) autologous chondrocytes can provide a non-immunogenic coating, preventing the foreign body reaction elicited by Medpor^®^; (2) engineered cartilage can possess various physical properties based on the choice of inert materials; and (3) possible capsule formation or intrusion, or tissue necrosis can be minimized by the inert material.

In the previous study, Medpor^®^ implant was attempted to be coated with gel-type materials such as fibrin gel and Pluronic F-127 mixed with cells, and the outcome was somewhat optimistic [21,22]. Even distribution of chondrocytes may induce a synergistic effect of cell-to-cell communication, and soft bed of the gel-type materials can help to deposit cartilage-specific ECM to increase regeneration potential. A downside of the use of fibrin gel or Pluronic F-127 is that they limit the construct’s applications, as the materials are weak in structural property and difficult to shape or sustain. Subsequently, the outcome of engineered cartilage in this way indicted that only one side of Medpor^®^ implant was covered with cartilage tissues and the other side was disturbed during in vivo implantation. In most tissue engineering applications, fibrin is introduced through injection. However, if these polymers can be successfully converted to stiff solid polymers in multiple ways, including crosslinking and bonding reinforcement, in the near future, gel-type of materials in cartilage tissue engineering application will be a strong candidate for overcoming their weak structural properties.

Conversely, PLGA can provide adequate structural integrity without early deterioration.

This study focused on the proof of concept by using a small scale of samples to test the feasibility of regenerating the whole structure of the human ear or nose cartilage on a larger scale of regeneration in the future. However, we utilized autologous primary chondrocytes as the cell source to regenerate neocartilage, which is a therapeutic model in the clinical setting. As an alternative option for autologous cell source, current advances in stem cell technology may offer various choices of stem cell sources that can differentiate into chondrogenic lineage cells, including mesenchymal stem cells (MSCs), induced pluripotent stem cells (iPSCs), embryonic stem cells (ESCs), and amniotic stem cells (ASCs), demonstrating comparable differential potential to chondrogenic lineage similar to primary chondrocytes [23,24,25,26]. The major advantage of using stem cells is the prevention of apoptosis of the primary chondrocytes after over passaging for the expansion, whereas there are risks of tumorigenesis, rejection, viral vector issue, and so on.

The monolayer expansion of autologous primary chondrocytes usually dedifferentiate, and this limits its applicability in large-scale cartilage tissue engineering. As an alternative use for stem cells, co-culturing of both autologous chondrocytes and MSCs together would be an option in the pursuit of the shortage of an autologous cell source. Cohen et al. demonstrated the successful outcome of patient-specific human ear cartilage engineered exclusively with human cell sources without extensive in vitro cell expansion prior to implantation. This study was a critical step towards the clinical application of tissue engineering for ear cartilage reconstruction [27].

A key to successfully engineering cartilage is homogeneously distributing chondrocytes throughout the PLGA scaffold. Static seeding techniques are the most frequently used method, as they are easy to use and have less chance of damaging cell by external force. However, additional studies on seeding will be useful because the optimal seeding method can increase seeding efficiency, allowing a higher number of cells in the seeding suspension to adhere to the scaffold. It would also be reproducible and easy to perform, leading to consistent outcome. In fact, our early outcome for the engineered tissue with PLGA and PLGA–Medpor^®^ did not form much cartilage-like tissues, but instead formed mostly fibrous tissues. This might have been caused by the poor seeding method, which led to a lower cell seeding efficiency and less homogeneous distribution of the cells throughout the scaffold when compared with dynamic seeding techniques [28,29]. To overcome the limitation, we applied the vacuum after static seeding, and the result demonstrated greater efficiency of cell seeding in comparison to the static seeding counterpart. As a result, cartilage tissue regeneration was improved in a significant manner. Additional dynamic seeding may improve further in vitro maturation for better cartilage tissue regeneration in vivo. Nonetheless, we still could achieve satisfactory results with additional vacuum seeding only, which did make the procedure more concise for complete coverage of Medpor^®^ with tissue-engineered cartilage.

Recent 3D printing technology can provide an opportunity to combine different types of materials to engineer a complex structure of scaffold. Therefore, human ear shape scaffold, for example, can be printed using 3D printing technology in a more precise and controlled manner [30]. Furthermore, the broad selection of material with various physical properties and controlled pore size distribution and pattern provided by 3D printing technologies can allow more advanced, functional, durable, and patient-specific cartilage tissue [31].

It is concluded that cartilaginous tissue can successfully regenerate around a Medpor^®^ implant. Although we demonstrated engineered cartilage using PLGA–Medpor^®^ scaffold in a small scale in this study, we will move on to a larger, whole ear or nose-size scale of implant material in the near future.

## 4. Materials and Methods

### 4.1. Chondrocyte Isolation

New Zealand white rabbit (Charles River Laboratories, Wilmington, MA, USA) was euthanized, and elastic cartilage was obtained from its ear. Animal studies were carried out according to the protocol approved by the Institutional Animal Care and Use Committee at the University of North Carolina at Chapel Hill (protocol no. 17-151). All procedures were performed under sterile conditions. The perichondrium was removed, and the cartilage was finely minced, and then enzymatically digested in 0.2% collagenase type II (Wartington Biochemical, Freehold, NJ, USA) solution at 37 °C with constant agitation. After 2 h, chondrocytes were separated by centrifugation and washed with 1× PBS. Isolated chondrocytes were gently mixed with chondrogenic media (DMEM containing 10% fetal bovine serum, 1% penicillin and streptomycin, ascorbic acid, and L-proline), plated in a 10 cm culture dish, and stored in incubator maintaining 5% CO_2_ at 37 °C for further expansion. The medium was changed every 3 days.

### 4.2. Detection of Collagen Type II Gene and Protein

RNA was isolated from the cultured chondrocytes (3 × 10^5^ cells) using an RNeasy kit (Qiagen, Chatsworth, CA, USA), and the total RNA was reverse-transcribed into cDNA by RT-PCR. Oligonucleotide primers for the PCR were designed according to the published sequence for the type II collagen (sense, GTG GTG AGA CTG GTG CTG TG; antisense, TGG TTG TTG AGG GAC TTG AG). The PCR conditions used for type II collagen production were 22 cycles of denaturation (95 °C/l min), annealation (60 °C/1 min), and extension (72 °C/1 min). The oligonucleotide primers for beta-2M were used as a control. Then, the sample was electrophoresed through a 2% agarose gel containing ethidium bromide.

For Western blot analysis, media were collected and denatured at 95°. The sample was separated on a 12% SDS-PAGE gel, transferred to an immobilon-P membrane (Millipore, Danvers, MA, USA), and visualized using an enhanced chemiluminescent substrate (Thermo Scientific, Waltham, MA, USA). Anti-collagen type II antibodies (Santa Cruz biotechnology Inc., Santa Cruz, CA, USA) were used to detect secreted collagen type II.

For immunostaining of type II collagen, chondrocyte culture was fixed in 4% paraformaldehyde solution (Sigma-Aldrich, St Louis, MO, USA) for 30 min. The cells were incubated with bovine anti-collagen II polyclonal primary antibody (ThermoFisher Scientific) diluted to 1:100 in 1% Bovine serum albumin (BSA) solution at 4 °C overnight followed by incubation with 3% BSA in 1×PBS for 30 min to block unspecific background staining. After washing with 1xPBS three times, Alexa Fluor Plus 488 (ThermoFisher Scientific) as secondary antibody was then applied for 30 min and then subjected to three more washes in 1xPBS, which was followed by treatment with propedium idodie (PI, 1 ug/mL; ThermoFisher Scientific) for nuclei staining. Images were acquired using Nikon Eclipse Ti-U inverted fluorescent microscope (Nikon Instruments Inc., Melville, NY, USA).

### 4.3. PLGA–Medpor^®^ Scaffold Preparation

Both PLGA and PLGA–Medpor^®^ scaffolds were fabricated as disk shapes. A cylindrical Teflon mold with a hole (100 mm in diameter and 10 mm in depth) in the middle was created. A total of 5 g PLGA (P2066: Sigma-Aldrich, St. Louis, MO, USA) with the ratio of PLA to PGA as 65:35 was dissolved in 10 mL CHCl_3_ and mixed with 40 g of salt (sized between 200 and 250 µm). To make the three-dimensional porous structure of the polymer scaffold, salt leaching methods were applied. Medpor^®^ (0.8 mm in thickness) was cut to be 8 mm in diameter. Dissolved PLGA with salt was poured into the mold, up to half of the mold volume; Medpor^®^ was placed in the mold; and then the remaining mixture was poured in to acquire the desired thickness. After complete drying, the mold was placed in distilled water to leach out salt, and was dehydrated and then sterilized with ethylene oxide gas. PLGA scaffold was made the same way, without Medpor^®^.

### 4.4. Cell Seeding and In Vivo Cartilage Regeneration

**Figure 6:**
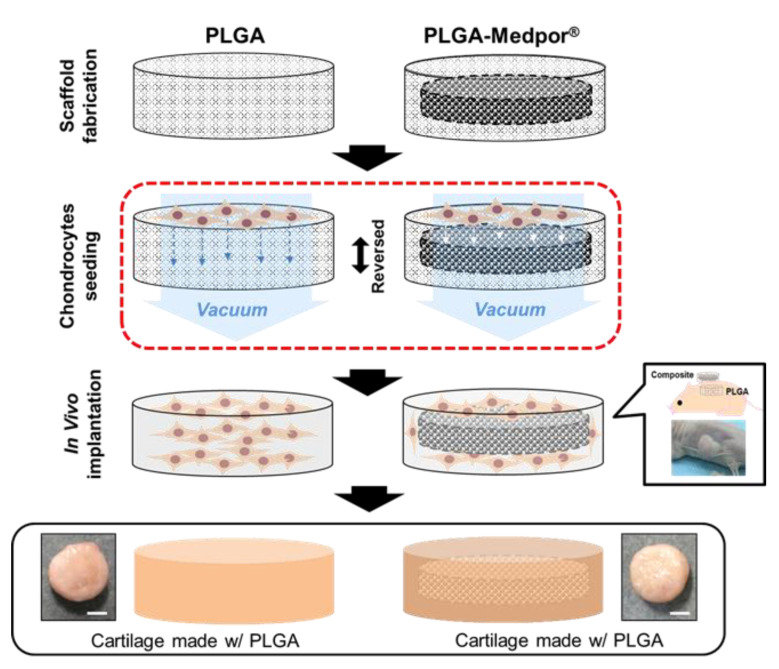
Vacuum cell seeding on PLGA and PLGA–Medpor^®^. After 12 weeks of implantation in the dorsal pocket of nude mice, neocartilage were formed around both PLGA and PLGA–Medpor^®^, demonstrated a solid morphology with milky color (Scale: 400 µm).

Chondrocytes were detached using 0.05% trypsin–EDTA and prepared as two batches of 5 × 10^6^ cell suspension in 25 µL media for each scaffold. Statically seeded scaffold was used to study the effect of vacuum. A sterile bottle top filter (ThermoFisher Scientific) was placed on a glass bottle and connected to a vacuum hose. After the vacuum ran on the surface of the filter, the level of vacuum was adjusted to slowly let 25 µL of media drop on the surface in order to get absorbed into the scaffold. After the scaffold was placed on the filter, vigorously mixed 25 µL of cell suspension was seeded on top of the scaffold surface using a pipette. The scaffold was quickly removed from the filter once the cell suspension was absorbed into the scaffold and was placed in growth media for least 2 h. The same procedure was repeated on the other side of the scaffold (Figure 6). Each scaffold was placed in each well of a 24-well plate containing growth media. After 24 h, the scaffolds were implanted subcutaneously, under the dorsal skin of nude mouse (4 weeks, balb/c; Charles River, Wilmington, MA, USA). Animal studies were carried out according to the protocol approved by the Institutional Animal Care and Use Committee at the University of North Carolina at Chapel Hill (protocol no. 17-151). To implant the scaffold, animals were anesthetized using isoflurane, an incision about 1.5 cm in size was made on the middle of the back, and a pocket was created by opening the incision with the back of scissors. Cell-seeded PLGA scaffold and the composite scaffold were carefully inserted using forceps on the left and right sides of the back, respectively. The skin opening was closed by suturing with 4-0 polypropylene (Johnson and Johnson, New Brunswick, NJ, USA). Nude mice were returned to their cage and managed by vet care for 12 weeks.

### 4.5. Scanning Electron Microscopic Analysis

After 12 weeks of implantation, the implants were harvested and fixed in 2.5% glutaraldehyde (Gibco Laboratories, Grand Island, NY, USA) for 2 days at room temperature. After thorough washing with PBS, the explants underwent critical point drying process by dehydrating through an ethanol gradient (35%, 50%, 70%, 80%, 95%, and 100% ethanol for 10 min each) and drying in hexamethyldisilazane (HMDS; Sigma, MO). Subsequently, the cross-sectional surfaces of the samples were sputter-coated with platinum at thickness of 4 nm in a vacuum and were examined using scanning electron microscopy (SEM; Model S-2256N, Hitachi, Naka, Japan).

### 4.6. Histological Analysis

Each sample was harvested 12 weeks after implantation, and fixed in 10% neutral buffered formalin solution for 2 days. After dehydrating in 95% and 100% ethanol sequentially, the samples were transferred to the tissue processor for paraffin embedding. Then, the blocks were sectioned at 5 μm, deparaffinized with xylene, and stained with Weigert’s hematoxylin (Sigma-Aldrich, Saint Louis, MO, USA) and counter-stained with eosin (Sigma-Aldrich) to assess cell morphology. Masson’s trichrome and Verhoeff staining were conducted using ready-to-use kits following the company’s instruction. Briefly, for the Masson’s trichrome staining (Sigma-Aldrich, Saint Louis, MO, USA), slides were immersed in Bouin’s solution for 15 min. Subsequently, the slides were stained with Weigert’s hematoxylin for 5 min and with Biebrich scarlet acid fuchin for 5 min. The stained slides were differentiated in phosphotungstic/phosphomolybdic acid solution for 5 min to enhance the staining of the collagen. Finally, the samples were dyed with aniline blue solution for 5 min and differentiated in 1% acetic acid solution for 2 min to avoid color removal. Verhoeff staining was carried out using Elastic Stain Kit (American MasterTech Scientific, Lodi, CA, USA). Slides were stained in Verhoeff’s solution for 60 min and then differentiated in 2% ferric chloride for 1 to 2 min after rinsing in tap water. The differentiation was stopped by immersing the slides in tap water after checking microscopically for black elastic fiber. The counterstaining in hematoxylin solution was carried out for 5 min. Then, slides were mounted and neocartilage formation was analyzed using a Nikon Eclipse Ti-U inverted microscope (Nikon Instruments Inc.). Collagen and elastin were quantified in as percentages using ImageJ software [32].

### 4.7. Mechanical Analysis

The compressive strengths of the explants made with PLGA and PLGA–Medpor^®^ were tested using Instron (ElectroPuls E3000, Canton, MA, USA). The explants were stored in PBS until the compression test was performed. Specimens were compressed by 0.5 mm/min uniaxial force. Five specimens were tested per each group. The maximum strength of each specimen was determined from the stress–strain curve.

### 4.8. Statistics

One-tailed Student’s *t*-test was used to compare the means between the groups.
